# The Difference in the Assessment of Knee Extension/Flexion Angles during Gait between Two Calibration Methods for Wearable Goniometer Sensors

**DOI:** 10.3390/s24072092

**Published:** 2024-03-25

**Authors:** Tomoya Ishida, Mina Samukawa

**Affiliations:** Faculty of Health Sciences, Hokkaido University, North 12, West 5, Kita-ku, Sapporo 060-0812, Japan; t.ishida@hs.hokudai.ac.jp

**Keywords:** gait analysis, knee kinematics, motion analysis, wearable sensor, validation

## Abstract

Frontal and axial knee motion can affect the accuracy of the knee extension/flexion motion measurement using a wearable goniometer. The purpose of this study was to test the hypothesis that calibrating the goniometer on an individual’s body would reduce errors in knee flexion angle during gait, compared to bench calibration. Ten young adults (23.2 ± 1.3 years) were enrolled. Knee flexion angles during gait were simultaneously assessed using a wearable goniometer sensor and an optical three-dimensional motion analysis system, and the absolute error (AE) between the two methods was calculated. The mean AE across a gait cycle was 2.4° (0.5°) for the on-body calibration, and the AE was acceptable (<5°) throughout a gait cycle (range: 1.5–3.8°). The mean AE for the on-bench calibration was 4.9° (3.4°) (range: 1.9–13.6°). Statistical parametric mapping (SPM) analysis revealed that the AE of the on-body calibration was significantly smaller than that of the on-bench calibration during 67–82% of the gait cycle. The results indicated that the on-body calibration of a goniometer sensor had acceptable and better validity compared to the on-bench calibration, especially for the swing phase of gait.

## 1. Introduction

Healthy life expectancy is an important estimate of how long people can enjoy a state of good health, and enhancing healthy life expectancy is a challenge for society. Maintaining mobility is a critical factor for healthy life expectancy and health-related quality of life among elderly individuals [1,2,3]. Locomotive disability and the risk of falls are closely linked to lower-extremity kinematics during a gait, and thus the assessment of gait kinematics during daily living is of increasing interest in the field of health promotion [4,5,6]. However, the conventional method for assessing lower-extremity kinematics relies on an optical three-dimensional motion capture system, which is typically limited to a laboratory setting and requires elderly individuals and/or patients to visit the laboratory. Moreover, such a system requires a time-consuming data acquisition and analysis process.

Several alternative methods to evaluate gait kinematics have been investigated. Recently, gait analysis systems using single or multiple video cameras and human pose estimation artificial intelligence have also attracted attention [7,8,9,10,11]. These systems have the advantage of not requiring anything to be attached to the subject, but these systems have a limited angle of view for cameras. In addition, there is a restriction that people other than the subject should not be shown (for analysis and privacy reasons). Therefore, gait assessment using video cameras and pose estimation has limitations for assessment in daily life. Cadence and step time variability of gait differ between laboratory and daily-life assessments [12], and gait analysis in daily life is expected to become more important for health care and health promotion [13].

Wearable sensors can be another alternative method to evaluate gait kinematics in daily life and are increasingly being used for the evaluation of lower extremity kinematics [13]. The assessment using these sensors can be conducted anywhere and is not limited to a space, and the data are easier to record and analyze than traditional optical motion capture systems [13]. Inertial measurement units (IMUs) with accelerometers and gyroscopes are one of the most commonly used wearable systems for kinematic analysis. IMUs are attached to each body segment and can analyze the three-dimensional kinematics of lower extremity joints. Moreover, spatio-temporal parameters such as gait speed, step length, and cadence can also be assessed [6,14]. However, some calibration procedures are required to convert the sensor coordinate to the anatomical coordinate [15,16]. The addition of a magnetometer sensor provides accurate orientation of the sensors in space and has shown good repeatability of calibration [17], while magnetometer sensors are affected by the magnetic field [13,18]. In addition, these systems require some processing to remove drift due to integral calculations, which limits their ability to measure over long periods of time [19].

Wearable electro-goniometers, another common wearable system, can measure joint angles during gait. Electro-goniometry is one of the oldest methods used to evaluate knee extension/flexion motion during gait and has been reported since the 1970s [20]. A single independent electro-goniometer unit is attached to each joint. Electro-goniometer is free of drift or magnetic field. Measurements and calculations are simpler and less cumbersome than motion capture systems using multiple IMUs. In recent years, lightweight wearable electro-goniometers have been developed that can be controlled and analyzed using a smartphone application, and real-time visual biofeedback can be obtained [21,22]. Therefore, wearable goniometers have great potential for use in telehealth to facilitate rehabilitation effects after injury and are expected to be used in health promotion services that visit the elderly or local communities. However, the measurement errors of wearable goniometers for assessing lower-extremity kinematics have been reported to be greater than those of IMU-based wearable systems, which is a cause for concern [18].

Measurement of knee joint angle using an electrical goniometer is affected by the skeletal alignment of the lower extremity, especially in the knee flexed position [18,21,23]. The knee joint is not a pure hinge joint (a degree of freedom) and exhibits axial rotation and anterior/posterior translation during knee extension/flexion, even for active motion in the absence of external forces [24]. The knee adduction/abduction angles also change with the knee flexion angle even under unloaded conditions [25]. These frontal and axial knee motions can affect the accuracy of the electrical goniometer due to kinematic crosstalk when assessing the knee flexion angle during gait [23]. Therefore, goniometer calibration that accounts for the secondary motion associated with knee extension/flexion for each individual may be important to reduce kinematic crosstalk due to secondary knee motion.

Recently, we reported acceptable accuracy (absolute error <5°) of a wearable goniometer sensor for assessing knee extension/flexion angle during gait [22], which is comparable to gait analysis using IMU systems [26] and human pose estimation [7,8,11]. We hypothesized that calibrating the goniometer on an individual’s body would reduce errors compared to calibrating the goniometer on a bench [22]. However, the previous study did not compare the errors between on-bench and on-body calibration methods. In addition, the errors occurring during the swing phase were larger than those occurring during the stance phase [22]. Calibration of the goniometer in our previous study was performed in the standing position and the preferred sitting positions of participants. Therefore, we also hypothesized that the calibration angle in the sitting position may be too small to evaluate the knee flexion angle during the swing phase of gait [22]. Since the maximum knee flexion angle during the swing phase is ~70°, calibration at a larger angle may reduce the error during the swing phase.

In summary, wearable goniometers have several advantages for the assessment of knee extension/flexion motion during gait and potential for use in daily life, while some disadvantages, especially in terms of accuracy, should be addressed (Table 1). The purpose of the present study was to compare the accuracy of knee flexion angle assessment during gait using a wearable goniometer calibrated on an individual’s body with one calibrated on a bench with an optical motion analysis system. In addition, we specified a calibration angle of 90° to reduce errors during the swing phase. It was expected that the on-body calibration of the goniometer sensor would have a more similar waveform and less deviation from the three-dimensional optical motion analysis than the on-bench calibration of the goniometer sensor, especially during the swing phase of the gait cycle.

## 2. Materials and Methods

### 2.1. Participants

Ten young adult participants (five males and five females; mean ± SD (range), age 23.2 ± 1.3 years (21–25 years), body height 164.0 ± 8.9 cm (151.9–178.2 cm), body mass 54.9 ± 6.5 kg (46.2–63.2 kg), body mass index 20.4 ± 1.3 kg/m^2^ (18.0–21.8 kg/m^2^)) were enrolled in this study. A prior sample size calculation showed that at least 7 participants were needed to achieve an alpha level (*α*) of 0.05, statistical power (1 − *β*) of 0.80, and a correlation coefficient of 0.8. The assumption of this correlation coefficient was based on our previous report investigating the correlation relationship of the knee flexion angles between the wearable goniometer sensor and a three-dimensional optical motion analysis system [22]. Participants were excluded from the present study if they reported knee pain during daily activity, any history of lower-extremity surgery, obvious deformities of lower-extremity, or any conditions that affect gait, such as neurological disorders. Written informed consent was obtained from participants before experiments, and this study was approved by the Institutional Review Board of the Faculty of Health Sciences, Hokkaido University (approval number: 21–74).

### 2.2. Procedures

**Figure 1:**
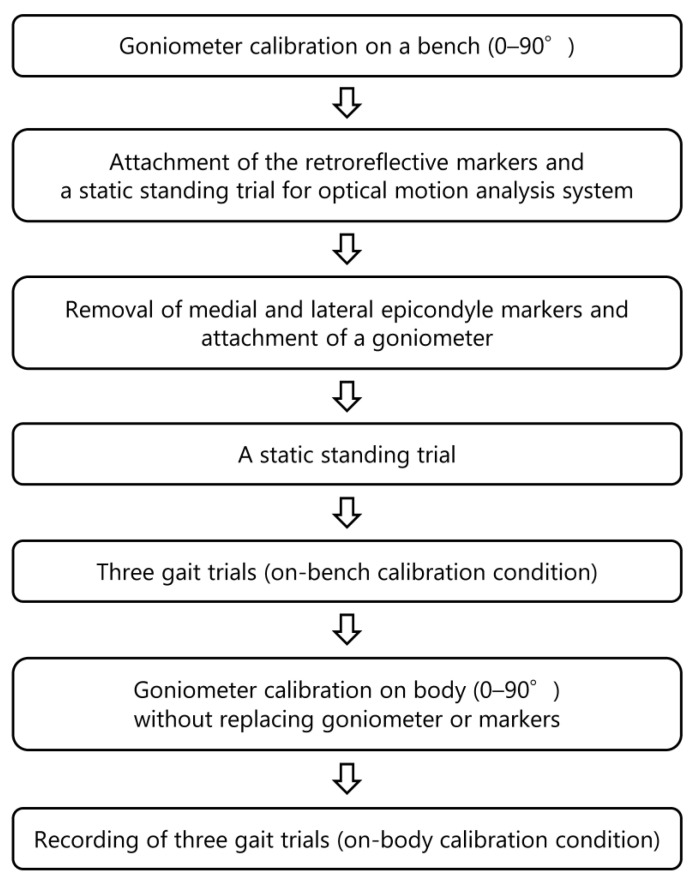
Experimental protocol.

**Figure 2:**
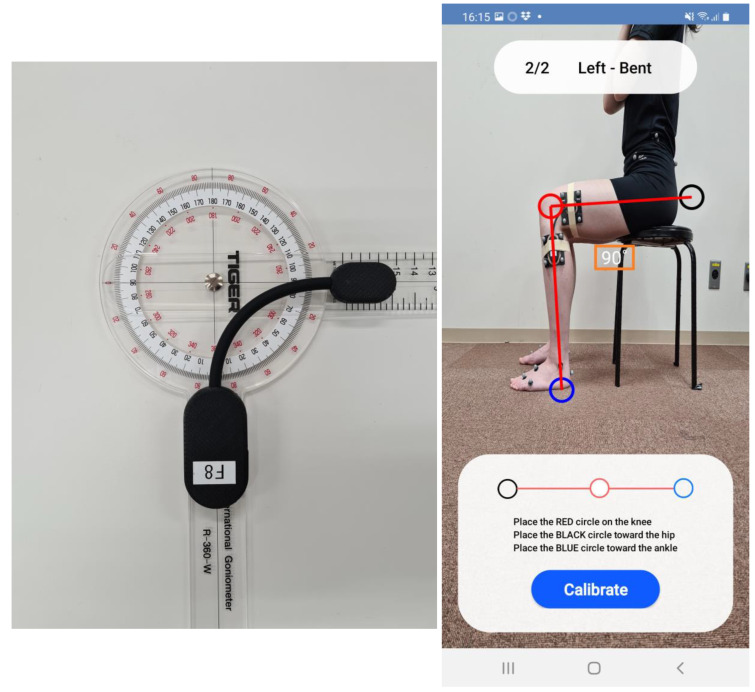
On-bench and on-body calibrations. In both calibration methods, knee flexion angles of 0° and 90° were confirmed by a single examiner using a universal goniometer and registered on a mobile application. The application allowed the examiner to manipulate the black, red, and blue circles on the screen and to calibrate at any two angles (the knee in extension and flexion), which in this study were defined as 0 and 90°.

To compare knee flexion angles between the two calibration methods, experiments were performed as shown in Figure 1. The same calibration angles of 0° and 90° were used for both calibration methods. First, a wearable goniometer was calibrated on the bench from 0° to 90°(Figure 2). Second, retroreflective markers for optical motion analysis were attached to participants, and a static standing trial was recorded to create each participant’s model for the optical motion analysis system. The medial and lateral epicondylar markers were then removed, and the goniometer sensor calibrated on the bench was attached. After some practice gait trials, three gait trials were recorded on a 6 m walkway using the optical motion analysis system and the wearable goniometer sensor as the on-bench calibration condition. The walking speed was selected by the participant. Next, the wearable goniometer was recalibrated with a range from 0° to 90° on the participant’s body (Figure 2). Markers and the wearable goniometer were not removed or reattached between the two calibration conditions. Three gait trials were recorded using the optical motion analysis system and the goniometer sensor as the on-body calibration condition in the same manner as in the on-bench calibration condition. All retroreflective marker attachments, wearable goniometer sensor attachments, and goniometer calibrations were performed by a single examiner.

### 2.3. Knee Kinematics Analysis Using a Wearable Goniometer

The wearable goniometer sensor (Smart Knee, Nitto Denko Corporation, Osaka, Japan) used in this study has a single axis and is based on a flexible bending sensor system. This goniometer is designed to monitor knee flexion motion during daily living activities such as gait. It weighs 37.4 g and is capable of approximately 6-day measurements with a button battery (CR2450, 260 mAh). The repeatability and output resolution of the goniometer sensor are less than 1.0° and 0.1°, respectively. Our previous study showed a high test–retest reliability for the assessment of knee extension/flexion motion during gait [22]. The coefficient of multiple correlations (CMC) was 0.988, and the average absolute error (AE) during the entire gait cycle was 2.5°, indicating a good to acceptable error [27,28]. The intraclass correlation coefficients (ICC) of peak knee extension/flexion angles were 0.880–0.927, indicating excellent reliability, except for peak knee flexion during the swing phase (ICC = 0.713, good reliability) [29]. The goniometer was attached to the thigh and shank marker clusters on the lateral aspect of the left knee using double-sided tape so that the center of the goniometer was aligned with the lateral femoral epicondyle, frequently referred to as the knee extension/flexion axis [30]. An experienced physiotherapist palpated bony landmarks (greater trochanter, lateral femoral epicondyle, and lateral malleoli) to align the goniometer with the lower extremity axis. Additionally, elastic taping was used to secure the goniometer to the thigh and shank to prevent rotation. The wearable goniometer was operated using a customized mobile application (SK App, Nitto Denko Corporation) via Bluetooth connection. The goniometer sensor was calibrated to two positions (knee in extended and flexed positions). Using the mobile application, a photograph was taken of each calibration position, and the knee flexion angle was measured on the application (Figure 2). In the present study, two calibration positions were set at 0° and 90° for both on-bench and on-body calibrations. A single examiner (an experienced physiotherapist) confirmed the knee flexion angles of 0° and 90° using a universal goniometer and registered them on the mobile application. The knee flexion angles during the gait trials were calculated by the mobile application using a linear fitting coefficient obtained from the two calibration positions (i.e., 0° and 90°). The sampling rate of the goniometer sensor was set at 200 Hz. A fourth-order Butterworth lowpass filter with a 6 Hz cut-off frequency was used to smooth the data at post-processing using MATLAB (MathWorks, Natick, MA, USA).

### 2.4. Knee Kinematics Analysis Using a Three-Dimensional Motion Analysis System

Retroreflective markers were attached to the pelvis and lower extremities based on a previous study [31]. Markers were placed on the iliac crest, anterior and posterior superior iliac spines, greater trochanters, medial and lateral femoral epicondyles, medial and lateral malleoli, second metatarsal head and base, and fifth metatarsal head and heel. In addition, thigh and shank marker clusters were attached to the lateral aspect. The greater trochanter marker was only used to align the goniometer with the lower extremity axis and was not used for three-dimensional motion analysis. The lateral femoral epicondyle markers were removed after recording the static standing trial to avoid interference with the goniometer. The medial femoral epicondyle markers were also removed because of contact on both sides during gait. Thigh and shank motions during gait trials were tracked using thigh and shank marker clusters.

**Figure 3:**
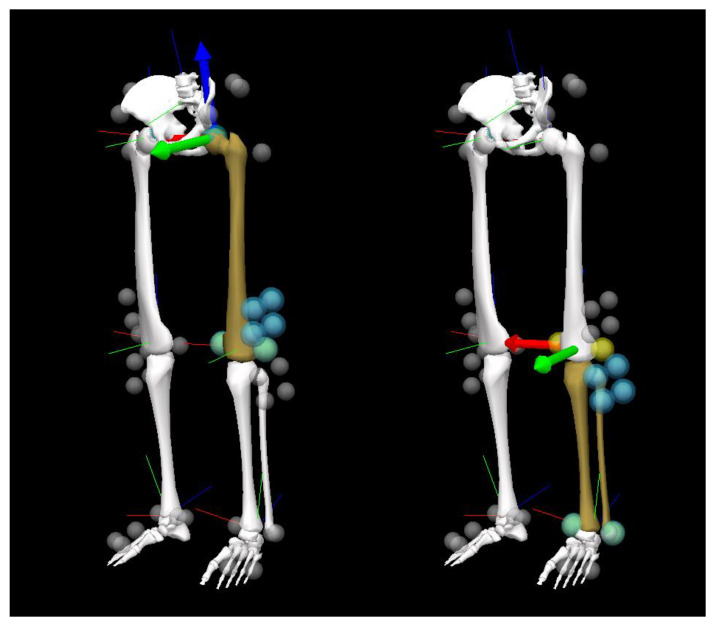
Thigh (**left**) and shank (**right**) segment coordinate systems in the optical motion analysis systems. The red, green, and blue axes indicate *X*-, *Y*-, and *Z*-axes, respectively.

Marker trajectories were recorded using a three-dimensional motion analysis system (Cortex version 5.0.1, Motion Analysis Corporation, Santa Rosa, CA, USA) with seven cameras (Hawk cameras, Motion Analysis Corporation). First, static calibration was performed using an L-shaped frame with four retroreflective markers to define the laboratory coordinate system. Next, a dynamic camera calibration was performed using a T-shaped wand with three retroreflective markers to calibrate the cameras. The result of camera calibration showed that the mean and standard deviation of the measurement error for the wand at 500 mm distance were <0.1 mm and <1 mm, respectively. The marker trajectory data were sampled at 200 Hz. The knee flexion angle was calculated using Visual3D software (version 6, C-Motion, Inc., Germantown, MD, USA). Marker trajectories were lowpass filtered using a fourth-order Butterworth filter with a 6 Hz cut-off frequency. The femur (thigh) coordinate system was defined using the medial/lateral femoral epicondyle markers and the hip joint center (Figure 3) [22]. The hip joint center position was calculated using the anterior/posterior superior iliac spine markers [32]. The X-axis (mediolateral) of the femur coordinate system was defined as the direction from the medial femoral epicondyle to the lateral femoral epicondyle. The Z-axis (inferior-superior) of the femur coordinate was defined as the direction from the midpoint of the medial and lateral femoral epicondyle to the hip joint center. The Y-axis (anteroposterior) of the femur coordinate was oriented forward, perpendicular to the X- and Z-axes. The tibia (shank) coordinate system was defined using the medial/lateral femoral epicondyle markers and medial/lateral malleoli markers (Figure 3) [22]. The X-axis (mediolateral) of the tibia was defined as the direction from the medial malleoli to the lateral malleoli. The Z-axis (inferior-superior) of the tibia was defined as the direction from the midpoint of the medial and lateral malleoli to the midpoint of the medial and lateral femoral epicondyle. The Y-axis (anteroposterior) of the tibia coordinate system was oriented forward, perpendicular to the X- and Z-axes. The knee flexion angle was calculated using a joint coordinate system with the Cardan *X-Y-Z* rotational sequence (extension/flexion, adduction/abduction, and then internal/external rotation) [33,34].

### 2.5. Data Analysis

Initial contact (IC) was defined as the time at the peak knee extension during the terminal swing since the purpose of this study was to investigate the difference between measurements obtained by the wearable goniometer and three-dimensional motion analysis. The knee flexion angle was normalized to 101 data points (i.e., 0 to 100%) for a gait cycle (IC to the next IC) for both the goniometer measurements and three-dimensional motion analysis. Due to the limited field of view of the three-dimensional optical motion analysis system, one gait cycle was analyzed per gait trial. In the present study, the angle in the static standing trial was offset to neutral (i.e., zero degrees) for both the wearable goniometer and the three-dimensional motion analysis system in order to compare the angle measured by both systems [22]. The absolute error between the wearable goniometer system and the three-dimensional optical motion analysis system was calculated for every 1% of the gait cycle. In addition, mean absolute error (AE) throughout the gait cycle was also calculated. The mean of three trials for each condition and each system was used in the statistical analysis. This processing was performed using MATLAB.

### 2.6. Statistical Analysis

All data are presented as the mean and standard deviation (SD). The knee flexion angle waveforms were compared between the wearable goniometer and optical motion analysis system using CMC for comparison of different systems [35], a one-dimensional statistical parametric mapping (SPM) paired *t*-test [36], and AE. Since there are cases where the CMC is low (weak correlation) even when the error is small and cases where the CMC is high (strong correlation) even when the error is large [27], both CMC and AE were assessed in this study. The CMC was interpreted as follows [37]:

- CMC ≥ 0.95: excellent
- 0.95 > CMC ≥ 0.85: very good
- 0.85 > CMC ≥ 0.75: good
- 0.75 > CMC ≥ 0.65: moderate

SPM analysis was performed using the open-source SPM 1d code (https://spm1d.org/index.html (accessed on 5 February 2021)) in MATLAB. AE was interpreted as follows [27,28]:

- AE ≤ 2°: good accuracy
- 2 < AE ≤ 5°: acceptable accuracy
- 5° < AE ≤ 10°: tolerable accuracy
- AE > 10°: unacceptable accuracy

Additionally, AE was compared between the on-bench and on-body calibration conditions using a one-dimensional SPM paired *t*-test. All statistical analyses were performed using MATLAB. The statistical significance level was set at *p* < 0.05.

## 3. Results

### 3.1. Waveform Similarity

The mean CMC was 0.993 (0.007) between the wearable goniometer with on-body calibration and three-dimensional motion analysis and 0.956 (0.029) between the wearable goniometer with on-bench calibration and three-dimensional motion analysis. All participants showed excellent similarity in the on-body calibration (range: 0.990–0.998), while six participants showed excellent similarity and four participants showed very good similarity in the on-bench calibration (range: 0.905–0.992) (Table 2).

### 3.2. Absolute Errors

**Figure 4:**
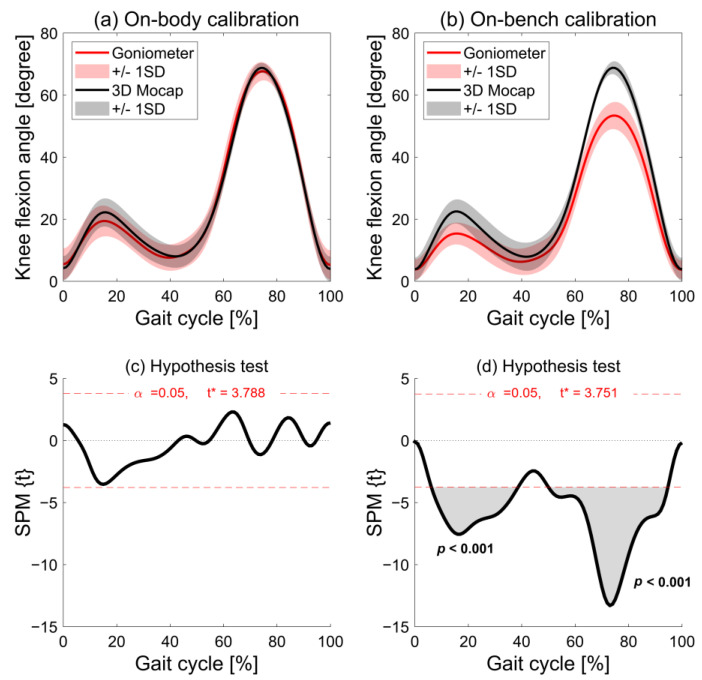
Comparison of knee flexion angles during a gait cycle acquired by three-dimensional motion analysis and wearable goniometer sensor with on-body calibration (**a**) and with on-bench calibration (**b**). The results of a statistical parametric mapping (SPM) paired *t*-test comparing the results of three-dimensional motion analysis and wearable goniometer sensor with on-body calibration (**c**) and with on-bench calibration (**d**). Red dashed lines in (**c**,**d**) indicate significant differences at *p* < 0.05. * indicates thresholds of significant difference.

**Figure 5:**
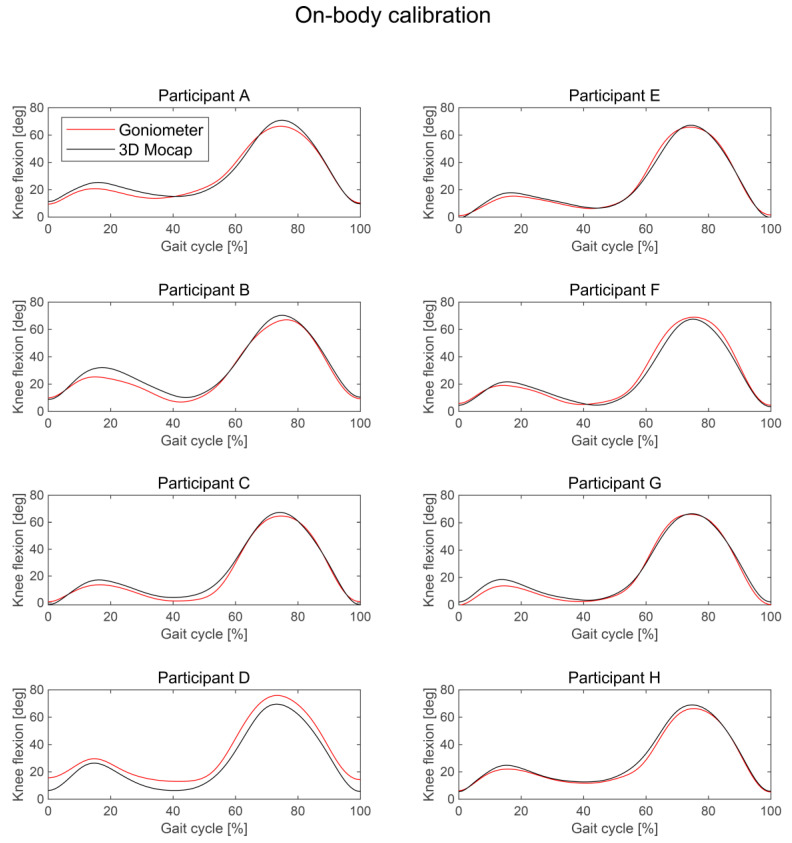
Comparison of knee flexion angles during a gait cycle acquired by three-dimensional motion analysis and wearable goniometer sensor with on-body calibration for each participant.

**Figure 6:**
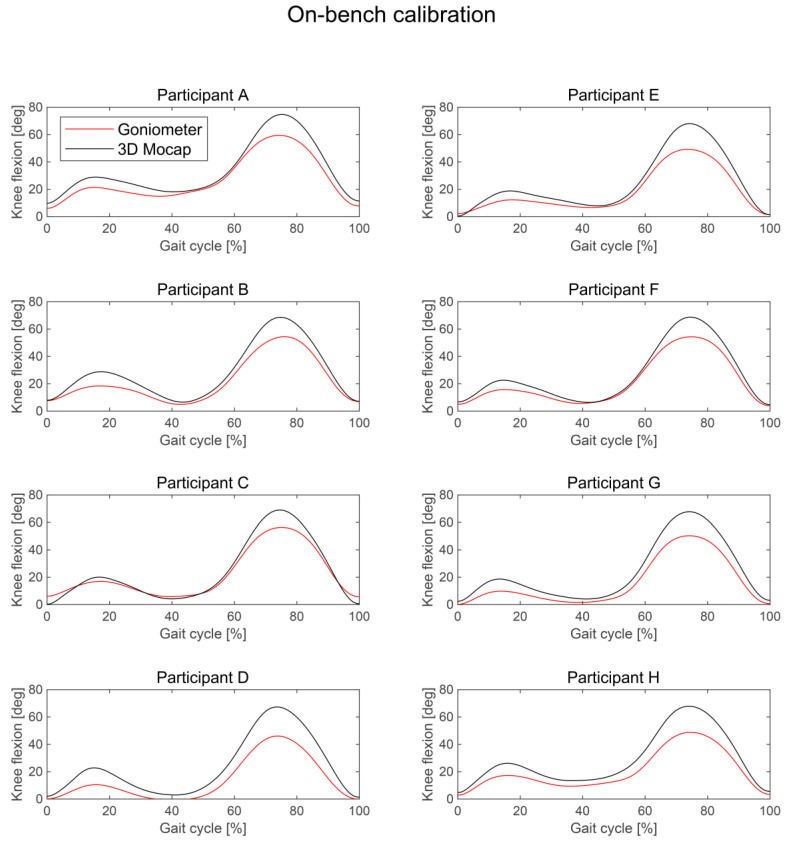
Comparison of knee flexion angles during a gait cycle acquired by three-dimensional motion analysis and wearable goniometer sensor with on-bench calibration for each participant.

SPM analysis showed that there was no significant difference between the knee flexion angle measured by the wearable goniometer with on-body calibration and the three-dimensional motion analysis throughout a gait cycle, indicating no significant fixed error (Figure 4a,c). The mean difference (SD) during the entire gait cycle was −0.2° (1.4°). The knee flexion angle during the stance phase tended to be smaller in the goniometer than in the motion analysis system, but the difference was not significant (Figure 4c). Except for this phase, there was no tendency for underestimation or overestimation evident when comparing the waveforms for each participant (Figure 5). On the other hand, the knee flexion angle measured by the wearable goniometer with on-bench calibration was significantly smaller than that measured by the three-dimensional motion analysis from 7 to 38% and 50% to 94% of the gait cycle (Figure 4b,d). The waveforms for all participants showed that the knee flexion angle measured by the goniometer with on-bench calibration was generally underestimated, especially for the peak knee flexion angle during both the stance and swing phases (Figure 6).

**Figure 7:**
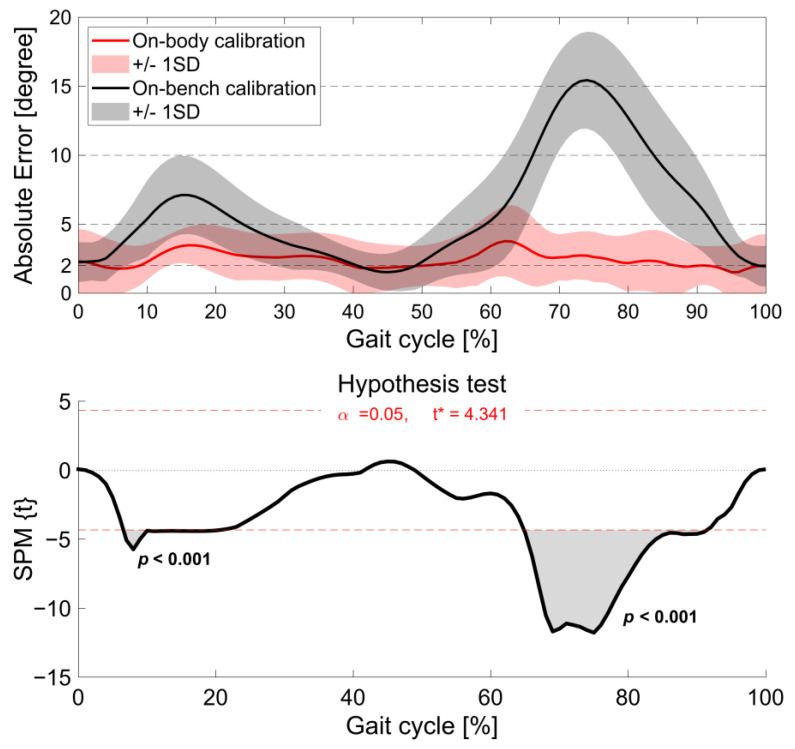
Comparison of the absolute errors (AEs) between results obtained using three-dimensional optical motion analysis and a wearable goniometer sensor with on-body calibration and on-bench calibration. Black dashed lines indicate the interpretation of AEs representing good (≤2°), acceptable (≤5°), tolerable (≤10°), and unacceptable (>10°) accuracy. Statistical parametric mapping (SPM) paired *t*-test results are shown with red dashed lines indicating statistical significance at *p* < 0.05. * indicates thresholds of significant difference.

The average AE during the entire gait cycle was 2.4° (0.5°) for the on-body calibration, and good (AE ≤ 2°) to acceptable accuracy (AE ≤ 5°) was observed throughout a gait cycle (range: 1.5–3.8°; Figure 7). The AEs during 3–8%, 40–50%, 87–90%, and 92–99% of the gait cycle were interpreted as having good accuracy. The average AE for the on-bench calibration was 6.0° (4.0°) (range: 1.5–15.4°). Unacceptable accuracy (AE > 10°) was observed from 67% to 83% of the gait cycle, tolerable accuracy (5° < AE ≤ 10°) was observed during 10–23%, 60–66%, and 84–92% of the gait cycle, and acceptable accuracy (AE ≤ 5°) was observed during 0–9%, 24–40%, 50–59%, and 93–98%. The other phases were considered as good accuracy (AE ≤ 2°). SPM analysis showed that AE was significantly smaller in the on-body calibration condition than in the on-bench calibration condition from 7% to 20% and 65% to 91% of the gait cycle (Figure 7). Regarding each participant’s data, four participants showed good mean AE, five participants showed acceptable mean AE, and one participant showed tolerable mean AE under the on-body calibration condition, while four participants showed mean acceptable AE, and the other six participants showed tolerable mean AE under the on-bench calibration condition (Table 3, Figure 5 and Figure 6). In comparison for each participant, larger errors were observed in knee flexion during the stance and swing phases for the on-bench calibration than for the on-body calibration.

## 4. Discussion

The present study showed that the knee flexion waveforms of a wearable goniometer with on-body calibration have excellent similarity to those of a three-dimensional motion analysis system. The SPM analysis and AE showed smaller errors for the wearable goniometer with on-body calibration than that with on-bench calibration in comparison with the three-dimensional motion analysis system. These findings indicate that the on-body calibrated wearable goniometer sensor has better validity in assessing knee flexion angles during gait than the on-bench calibrated sensor, which supports our hypotheses.

All participants showed excellent similarity for the on-body calibration (CMC ≥ 0.990), which is consistent with a previous study [19]. For the on-bench calibration, the mean CMC showed excellent similarity, but four participants showed very good similarity. The AE at IC (0% of the gait cycle), and during knee extension of the stance phase (24–50% of the gait cycle) was acceptable to good accuracy (AE ≤ 5°) for both the on-body and on-bench calibrations. However, the on-body calibration showed acceptable accuracy around the peak knee flexion during both the stance and swing phases, whereas the on-bench calibration showed tolerable to unacceptable errors (AE > 5°) during the phase (10–23% and 67–83% of the gait cycle) and significantly smaller knee flexion angles than the three-dimensional motion analysis (7–38% and 50–94% of the gait cycle). The SPM analysis also showed significant differences in AE during knee flexion in both stance and swing phases between the on-body and on-bench calibrations (7–20% and 65–91% of the gait cycle, respectively). Knee extension/flexion angles at IC, the peak knee flexion during the stance/swing phase, and the peak knee extension during the stance phase are commonly used in gait assessment for clinical and health promotion [6,38,39,40]. These results suggest that the wearable goniometer with on-body calibration has the potential to assess double knee action (two times of extension and flexion) during gait. On the other hand, the wearable goniometer with on-bench calibration has no practical utility in assessing the knee flexion angle during the stance and swing phases.

One of the sources of error in the measurement of knee flexion angle during gait using a wearable goniometer is secondary knee motion, which can include axial rotation, adduction/abduction, and anterior/posterior translation [18,21,23]. During gait, knee internal rotation motion occurs during the stance phase, whereas knee external rotation motion occurs during the swing phase [41]. Knee adduction/abduction motion also occurs during the swing phase [41]. Since goniometers are typically attached at the knee-extended position [42], the influence of secondary motion would be greater in the knee-flexed position (i.e., swing phase). Calibration in the flexed position under the on-body calibration condition partially accounts for each participant’s secondary knee motion. Therefore, the errors in the on-body calibration condition would have been smaller than those in the on-bench calibration condition, especially during the swing phase.

The mean AE of the present study using the wearable goniometer with on-body calibration (2.4°) was comparable to or better than that of the previous studies using IMU systems (root mean square error: 7.8°) [26] and human pose estimation (mean AE: 2.3–5.8) [7,8,11]. In addition, the AE of the wearable goniometer with on-body calibration seemed to be smaller than the results of a previous study using on-body calibration [22]. This previous study showed tolerable to good AEs (range: 1.3–6.2°), whereas the present study showed average AEs with acceptable to good accuracy throughout a gait cycle (range: 1.5–3.8°). These differences may be due to the difference in knee angle during flexed position calibration. Calibration in the knee-flexed position in the previous study was performed in the seated position with participants’ preferred knee angles, whereas the present study standardized the participants’ knee flexion angles to 90° in the seated position. A calibration angle of 90° would be better than that of a smaller angle when performing on-body calibration because the knee joint is flexed to approximately 70° during the swing phase.

Assessment of knee extension/flexion motion during the swing phase of gait is important for the elderly population with locomotive disorders [6]. Individuals with “locomotive syndrome” showed significantly smaller knee flexion angles and knee extension/flexion motion ranges during gait [6]. The knee flexion angle during the swing phase is also associated with toe clearance [40]. Toe clearance is smaller in elderly fallers than in non-fallers [43], and no difference was found between elderly and young adults [44]. Thus, assessment using a wearable goniometer sensor with on-body calibration is valuable in monitoring knee flexion during the swing phase in daily living to prevent locomotive disorders and falls. Gait analysis in the laboratory seems to reflect the individuals’ best performance [12], and assessments in the living environment using a wearable sensor could be important in predicting falls. In addition, patients with knee disorders such as osteoarthritis and anterior cruciate ligament injury showed altered knee extension/flexion motion during the stance phase [38,39]. A wearable goniometer with on-body calibration may also be useful in assessing such changes in knee extension/flexion motion before/after the rehabilitation and surgeries.

The present study had some limitations that should be acknowledged. This study included only healthy young participants. Therefore, these data may not be adequately validated for extrapolation to evaluate patients with gait disability and significant knee joint deformities, such as severe knee osteoarthritis. Future studies should clarify the validity of the wearable goniometer in populations with a wide range of ages, gait impairments, and/or significant knee joint deformities. Second, an experienced physiotherapist attached and calibrated the wearable goniometer sensor in this study. Simpler attachment and calibration methods need to be developed for practical use in telerehabilitation and health promotion in the elderly or in local communities. Third, although we did not replace the goniometer between the two calibration conditions in this study, there were potential errors due to the positioning variations of the wearable goniometer on the thigh and shank of the participant during gait. A simpler and more reliable fixation method should be investigated.

## 5. Conclusions

The present study demonstrated that the knee flexion waveforms of wearable goniometers with on-body calibration had excellent similarity (CMC ≥ 0.95) to those of optical three-dimensional motion analysis systems for all participants, while those with on-bench calibration had excellent similarity on average, but very good similarity for four participants (CMC ≥ 0.85). On-body calibration of the wearable goniometer showed no significant difference in the knee flexion angle from the three-dimensional motion analysis system in SPM analysis and had good (AE ≤ 2°) to acceptable accuracy (AE ≤ 5°) with a mean AE of 2.4° (0.5°) throughout the gait cycle, while on-bench calibration of the wearable goniometer showed a significant difference from the three-dimensional motion analysis during more than half of the gait cycle and had unacceptable (AE > 10°) to acceptable accuracy with a mean AE of 6.0° (4.0°). The AE of the wearable goniometer measurements were significantly smaller in the on-body calibration condition than in the on-bench calibration condition around the peak of knee flexion during the stance and swing phases (7–20% and 65–91% of the gait cycle).

The present study revealed that the on-body calibration of a goniometer sensor has acceptable and better validity for assessing knee extension/flexion angle during gait than on-bench calibration, especially during the swing phase of gait. On-bench calibration is not an appropriate method for gait assessment using a wearable goniometer. Future studies should evaluate the validity of wearable goniometers in populations with a wide range of ages, gait impairments, and/or significant knee deformities and explore simpler methods of attaching and calibrating the goniometer sensors for practical use in telehealth rehabilitation as well as health promotion.

## Figures and Tables

**Table 1:** General advantages and disadvantages of a wearable goniometer sensor for assessment of knee extension/flexion motion during gait.

Advantage	Disadvantage
Easy to use Single unit per joint Lightweight Controlled and analyzed using a smartphone application via blue-tooth connection Quick calibration	Less accuracy Sensitive to mounting position Affected by secondary motion (frontal/axial rotation and translation) Affected by calibration angle
Real-time visual biofeedback	Single or double axes (no 3D analysis)
No recording volume limitations	Mainly used for knee joint
Long time use (low power consumption, no drift)	No positional data
Less expensive (or cheap)	Requires attachment for sensors to body

**Table 2:** The coefficient of multiple correlations between the wearable goniometer sensor and optical system.

	Coefficient of Multiple Correlations	
Participant	On-Body Calibration	On-Bench Calibration
A	0.990	0.956
B	0.991	0.957
C	0.996	0.983
D	0.977	0.905
E	0.998	0.949
F	0.994	0.972
G	0.997	0.949
H	0.996	0.916
I	0.997	0.992
J	0.998	0.983
Mean	0.993 (0.007)	0.956 (0.029)

**Table 3:** Mean absolute errors between the wearable goniometer sensor and optical system for each participant.

	Mean Absolute Errors (SD) [min, max]	
Participant	On-Body Calibration	On-Bench Calibration
A	2.5 (1.3) [0.03, 4.7]	6.4 (4.1) [0.9, 15.5]
B	2.9 (2.1) [0.02, 7.5]	6.2 (4.0) [0.1, 14.7]
C	2.2 (1.3) [0.07, 5.2]	3.7 (3.5) [0.001, 12.7]
D	6.2 (1.6) [3.2, 9.5]	9.9 (5.9) [1.5, 21.3]
E	1.4 (1.1) [0.01, 4.9]	6.3 (5.4) [0.1, 14.4]
F	2.5 (1.6) [0.03, 6.9]	4.9 (3.9) [0. 2, 14.4)
G	1.9 (1.3) [0.004, 4.8]	7.6 (4.6) [2.2, 17.4]
H	1.7 (1.3) [0.02, 4.7]	8.3 (5.2) [1.8, 19.2]
I	2.0 (1.2) [0.06, 4.4]	2.7 (2.4) [0.01, 9.5]
J	1.1 (0.8) [0.01, 3.3]	3.5 (3.4) [0.001, 11.9]
Mean	2.4 (1.4)	6.0 (4.0)

## Data Availability

The datasets used and/or analyzed during the current study are available from the corresponding author upon reasonable request.
